# Molecular Cloning and Characterization of Sirtuin 1 and Its Potential Regulation of Lipid Metabolism and Antioxidant Response in Largemouth Bass (*Micropterus salmoides*)

**DOI:** 10.3389/fphys.2021.726877

**Published:** 2021-09-07

**Authors:** Yuting Huang, Shilin Wang, Xiaoxue Meng, Naisong Chen, Songlin Li

**Affiliations:** ^1^Research Centre of the Ministry of Agriculture and Rural Affairs on Environmental Ecology and Fish Nutrition, Shanghai Ocean University, Shanghai, China; ^2^National Demonstration Center on Experiment Teaching of Fisheries Science, Shanghai Ocean University, Shanghai, China

**Keywords:** sirtuin 1, characterization, lipid metabolism, antioxidant capacity, largemouth bass

## Abstract

Sirtuin 1 (SIRT1) of largemouth bass (*Micropterus salmoides*) was cloned and characterized in the present study and the influence of SIRT1 activation induced by resveratrol inclusion on the expression of genes related to lipid metabolism and antioxidation was also investigated. The SIRT1 of largemouth bass, with full-length cDNA sequence of 3395bp encoding 695 amino acids, was mainly expressed in gonad, heart and liver. The analysis of multiple sequence alignment revealed that, in accordance with other species, SIRT1 of largemouth bass contained highly conserved substrate-binding site and NAD^+^ binding site. The result of subcellular localization displayed that SIRT1 of largemouth bass was mainly localized in the nucleus. The inclusion of 1.0 and 2.5‰ dietary RSV, a natural SIRT1 activator, significantly elevated the SIRT1 protein expression. Meanwhile, the phosphorylation of AKT1 and FoxO1 followed similar pattern with that of SIRT1, indicating the activation of insulin pathway, which may result in the inhibition of lipogenesis and activation of lipolysis, and reduced hepatic triglycerides content. Additionally, the activation of SIRT1 induced by dietary RSV elevated the antioxidant capacity at both transcriptional level and enzymatic level, which was probably mediated by the transcription factor Nrf2. In above, SIRT1 was suggested to be involved in improving antioxidant capacity and alleviating hepatic lipid deposition in largemouth bass.

## Introduction

Sirtuin 1 (SIRT1), silent information regulator 2 (Sir2) homolog 1, is one of the seven mammalian homologs of yeast Sir2, which has been demonstrated to be involved in the regulation of some physiological process such as lipid metabolism (Simmons et al., 2015) and oxidative response (Chong et al., 2012). It has been demonstrated that SIRT1 can shuttle between nucleus and cytoplasm, and only nuclear morphology plays a regulatory role in the downstream target genes (Tanno et al., 2007; Pfister et al., 2008). Meanwhile, SIRT1 of mammals is mainly located in the nucleus (Houtkooper et al., 2012). The activation of SIRT1 can alleviate lipid deposition through inhibiting the expression of lipogenesis-related genes (Goldstein et al., 2002; Ponugoti et al., 2010) and promoting the rate of fatty acid oxidation (Lagouge et al., 2006; Feige et al., 2008), and the regulation process was partly mediated by the AKT (protein kinase B, PKB)/FoxO1 (Forkhead box O1) pathway (Tobita et al., 2016). Additionally, SIRT1 is involved in the regulation of antioxidant response by the nuclear factor E2-related factor 2 (Nrf2; Huang et al., 2013; Ding et al., 2016), which increases Nrf2-mediated gene expression such as superoxide dismutase (SOD), catalase (CAT), and glutathione peroxidase (GPx; Vnukov et al., 2017).

In practice, excessive lipid deposition and reduction of antioxidant capacity seriously restrict the sustainable development of aquaculture (Du, 2014). Therefore, it is essential to explore the regulation mechanism of lipid metabolism and antioxidant response in fish species. In teleosts, the decreased expression of SIRT1 in liver of gilthead seabream was accompanied with the down-regulation of oxidative metabolism and up-regulation of lipid synthesis (Simó-Mirabet et al., 2018). Additionally, the knockdown of SIRT1 in zebrafish was demonstrated to cause the oxidative injury and chronic inflammation (Kim et al., 2019). Thus, SIRT1 is suggested as a potential target for alleviating excessive lipid accumulation and oxidative stress in fish species.

Resveratrol (RSV) is a kind of non-flavonoid polyphenol with stilbene structure and widely existed in grape, peanut, mulberry and other seed plants (Burns et al., 2002). Recent study has shown that RSV can activate and increase the activity of SIRT1, and therefore, is considered as a natural SIRT1 activator (Borra et al., 2005). In mammals, the inclusion of RSV can activate SIRT1 and regulate hepatic lipid metabolism to improve hepatic steatosis (Baur et al., 2006; Zhou et al., 2018). It has been demonstrated that the antioxidant capacity of individuals with fatty liver was significantly reduced, and meanwhile, the decrease in antioxidant capacity aggravated lipid deposition (Videla et al., 2004). Also, RSV inclusion protects against from oxidative stress in mammals through the activation of SIRT1 (Li et al., 2013; Wang et al., 2020). The potential role of RSV on the regulation of lipid metabolism-related genes has been demonstrated in zebrafish (Ran et al., 2017) and blunt snout bream (Zhang et al., 2018), while the exact regulation mechanism remains unclear. Additionally, the inclusion of RSV significantly improves the antioxidant capacity of some fish species such as turbot (Tan et al., 2019) and blunt snout bream (Jia et al., 2019). Therefore, it is suggested that the physiological function of SIRT1 in the regulation of lipid metabolism and antioxidant capacity partly consistent with that in mammals.

Largemouth bass, *Micropterus salmoides*, has become one of the most important freshwater farmed fish because of its delicious taste and huge market demand. However, in production practice, excessive lipid accumulation induced metabolic liver disease and oxidative stress commonly impact the health status of this fish species. In view of potential molecular biology of SIRT1, this gene in largemouth bass was cloned and characterized in the present study. Meanwhile, RSV was supplemented to activate the expression of SIRT1 to determine its regulation of antioxidation and lipid metabolism in largemouth bass. It is envisaged that the findings of the present study will provide a new insight to attenuate abnormal lipid deposition and oxidative stress, and further promote the sustainable development of largemouth bass aquaculture.

## Materials and Methods

### Sequence Cloning

Total RNA was isolated from the liver of largemouth bass using the RNAiso Plus kit (TaKaRa, Japan). The first-strand cDNA synthesis of SIRT1 was accomplished using the isolated RNA as template following the instructions of PrimeScript^™^ RT reagent Kit (Takara, Japan). Partial cDNA fragments of largemouth bass SIRT1 cDNA were obtained with polymerase chain reaction (PCR) primers (Table 1), which was designed according to the protein coding region sequence of other fish SIRT1 using Primer 5.0. The primers were synthesized by Sangon Biotech (Shanghai) Co., Ltd (Shanghai, China). The PCR reaction conditions were set as follows: 94°C for 5min; 35cycles of 30s at 94°C, 30s at 54°C and 2min at 72°C; 72°C for 10min. After that, the amplified products were separated by 2% agarose gel electrophoresis, purified, ligated into the vector pEASY-T3 and sequenced to obtain the partial cDNA of largemouth bass SIRT1.

**Table 1:** Sequences of the primers used in this study.

Primer	Sequences (5'-3')	Primer information
SIRT1-F1	AAGATGGCGGACGGAGAGAG	RT primer
SIRT1-R1	ATTTAAAGGTGTGTGGTGCTC	RT primer
SIRT1-5-F1	CCCAGTCCATTGTTGTCTCCGCCT	5'RACE primer (inner)
SIRT1-5-F2	TCCTCAGTTGTTCCAAGCATAGCG	5'RACE primer (outer)
SIRT1-3-R1	CCATAGTGTGATGTCAGCTGTGGT	3'RACE primer (inner)
SIRT1-3-R2	ACGGCATACCATGAATTCAGCACT	3'RACE primer (outer)
SIRT1-F	TGGATTGTGAGGCTGTAAGG	qPCR
SIRT1-R	ATGAGGAATGGAGTTTGGGA	qPCR
Sirt1-Ecorl-F	GGATCCACTAGTCCAGTGTGGTGGAATGGCGGACGGAGAGAGC	Construction of expression plasmid
Sirt1-Ecorl-R	GCCACTGTGCTGGATATCTGCAGAAAAGGTGTGTGGTGCTCTGAGT	Construction of expression plasmid
Nrf2-F	CACCAAAGACAAGCGTAAG	RT-qPCR
Nrf2-R	GAAATCATCAACAGGCAGA	RT-qPCR
PPARα-F	CCACCGCAATGGTCGATATG	RT-qPCR
PPARα-R	TGCTGTTGATGGACTGGGAAA	RT-qPCR
Gpx-F	CTCCTCAACCAGGCAAAC	RT-qPCR
Gpx-R	ATACCCCCCTCACAACAA	RT-qPCR
CAT-F	TGAATGGCTATGGCTCTC	RT-qPCR
CAT-R	AATCTGGGTTGGTGGAAG	RT-qPCR
SOD1-F	TTATTTTGAGCAGGAGGG	RT-qPCR
SOD1-R	TTCTTGTTGTGGGGATTG	RT-qPCR
SOD2-F	GGTCTCATTCCCCTTCTT	RT-qPCR
SOD2-R	TCGCTCACATTCTCCCAG	RT-qPCR
ATGL-F	GCTCCCCTACACTCTCCCTCT	RT-qPCR
ATGL-R	CTGCTCTCGAATCCACTCAAC	RT-qPCR
ACO1-F	GGAGGTTATTGTTTCGGTTCT	RT-qPCR
ACO1-R	GCCTTCTTTTGGTCTTTTCTG	RT-qPCR
CPT1-F	GAATGGGGTAATGACTGGTGTG	RT-qPCR
CPT1-R	TCGACTGATTGGTATGTGTTGG	RT-qPCR
SREBP-1-F	TGGGGATTGGGCTATTCG	RT-qPCR
SREBP-1-R	CCTGGCTGGGGCTTTTCT	RT-qPCR
FAS-F	AGGCTGAGTGGGAGAAGGTG	RT-qPCR
FAS-R	GACGGCGACAAAGAAAGAGG	RT-qPCR
ACC1-F	ATCCCTCTTTGCCACTGTTG	RT-qPCR
ACC1-R	GAGGTGATGTTGCTCGCATA	RT-qPCR
ACC2-F	GACAGAGAGTGATTCAGGTGGA	RT-qPCR
ACC2-R	GCTGGTTGTTGGAAGTGTAGAC	RT-qPCR
β-actin-F	AAAGGGAAATCGTGCGTGAC	RT-qPCR
β-actin-R	AAGGAAGGCTGGAAGAGGG	RT-qPCR

The full-length cDNA sequence of SIRT1 was obtained by 5' and 3'-RACE PCR with the SMARTer™ RACE cDNA Amplification Kit (Clontech, CA, United States). Four specific primers (Table 2) were designed based on the obtained partial SIRT1 cDNA sequence. The two rounds of PCR were conducted according to the previously described method (Li et al., 2017). After that, the obtained PCR products were purified, ligated and sequenced to determine the corresponding sequences. Finally, the obtained sequences were spliced into the complete SIRT1 cDNA sequence for subsequent bioinformatics analysis.

**Table 2:** TG and lipoprotein content of largemouth bass fed diets with varying dietary RSV levels for 10weeks^*^.

	Dietary resveratrol content (g/kg diet)				
	0.0	0.5	1.0	2.5	5.0
Hepatic TG (mmol/gpro)	0.18 ± 0.02^a^	0.17 ± 0.00^ab^	0.13 ± 0.00^bc^	0.10 ± 0.02^c^	0.12 ± 0.00^c^
Serum TG (mmol/L)	5.14 ± 0.12^a^	4.64 ± 0.12^b^	4.19 ± 0.05^b^	4.16 ± 0.11^b^	3.42 ± 0.11^c^
LDL-C (mmol/L)	3.84 ± 0.10^b^	4.65 ± 0.06^a^	5.16 ± 0.14^a^	4.79 ± 0.17^a^	4.66 ± 0.12^a^
HDL-C (mmol/L)	3.69 ± 0.12^b^	3.42 ± 0.16^b^	4.48 ± 0.15^a^	4.02 ± 0.03^ab^	3.82 ± 0.15^b^
Cholesterol (mmol/L)	12.18 ± 0.09	12.03 ± 0.31	11.78 ± 1.02	11.71 ± 0.87	11.73 ± 0.33

* *Values (means ± SEM, N=3) within a row with a common superscript letter are not significantly different from the other dietary groups (p>0.05; Duncan’s test)*.

*TG, triglyceride; LDL-C, low density lipoprotein cholesterol; HDL-C, high density lipoprotein cholesterol*.

### Characterization of Full-Length SIRT1 cDNA

The BLAST program of NCBI^1^ was used to conduct homological analysis of largemouth bass SIRT1 nucleotide sequences, and the deduced amino acid (AA) sequence of the obtained SIRT1 was predicted with the software of DNAMAN 6.0. The functional domain of deduced AA was analyzed by NCBI conserved domains software.^2^ Multiple sequence alignment among largemouth bass SIRT1 and other orthologs, including *Sparus aurata* (AHX56273.1), *Salmo salar* (ENSSSAT00000005225.1), *Mus musculus* (NP_062786.1) and *Homo sapiens* (NP_036370.2), was performed with the Bioedit 7.0 software. The phylogenetic tree analysis was constructed using neighbor-joining method with the Mega 6.0 software.

### Subcellular Localization of SIRT1

The open reading frame (ORF) of largemouth bass SIRT1 without the termination codon was cloned with the primers in Table 3 and then ligated into restriction endonucleases XhoI enzyme-digested plasmid vector pCDNA3.1-EGFP using the pEASY-Basic Seamless Cloning and Assembly Kit (TransGen, Beijing). HEK293t cells were cultured in Dulbecco’s modified Eagle’s medium (HyClone) with 10% fetal bovine serum (Gibco) and 1% penicillin–streptomycin solution at 37°C in an aseptic incubator with 5% CO_2_. Then, the recombinant express vector was transfected into HEK293t using EZ Trans cell transfection reagent (Liji Biotechnology Co., Ltd., Shanghai). After the cells being cultured for 24h, they were fixed with 4% paraformaldehyde and labeled with DAPI, a nuclear dye, and then the localization being observed through a fluorescence microscope (Nikon, Eclipse80i). In addition, the ORF of Lamin A/B was cloned from HEK293t cells and then ligated into the vector pCDNA3.1-RFP as described above. After that, the expression vectors, pcDNA3.1-SIRT1-EGFP and pcDNA3.1-Lamin-RFP, were co-transfected into HEK293t cells to confirm the localization of SIRT1 with the fluorescence microscope (Nikon, eclipse80i).

**Table 3:** Antioxidant indexes of of largemouth bass fed diets with varying dietary RSV levels for 10weeks^*^.

	Dietary resveratrol content (g/kg diet)				
	0.0	0.5	1.0	2.5	5.0
SOD (U/mgprot)	173.78 ± 4.91^c^	190.65 ± 4.13^bc^	208.63 ± 2.9^ab^	227.78 ± 9.39^a^	218.44 ± 9.15^ab^
T-AOC (U/mgprot)	0.34 ± 0.03^c^	0.38 ± 0.02^c^	0.57 ± 0.03^b^	0.84 ± 0.05^a^	0.62 ± 0.01^b^
MDA (nmol/mgprot)	4.37 ± 0.35^a^	2.77 ± 0.10^b^	2.95 ± 0.11^b^	3.07 ± 0.17^b^	3.62 ± 0.04^ab^
CAT (U/mgprot)	8.94 ± 0.50^a^	5.53 ± 0.98^b^	4.67 ± 0.45^bc^	2.94 ± 0.11^c^	3.23 ± 0.14^bc^
H_2_O_2_ (nmol/mgprot)	19.99 ± 0.30^a^	21.28 ± 0.60^a^	18.52 ± 0.55^ab^	13.32 ± 0.92^c^	15.09 ± 1.24^bc^

* *Values (means ± SEM, N=3) within a row with a common superscript letter are not significantly different from the other dietary groups (p>0.05; Duncan’s test)*.

*SOD, superoxide dismutase; T-AOC, total anti-oxidative capacity; CAT, catalase; MDA, malondialdehyde*.

### Resveratrol Inclusion Study

The feeding trial was carried out under the recycling freshwater system following the standard operating procedures in the guide for the use of experimental animals of Shanghai Ocean University. Five isonitrogenous and isolipidic diets were formulated with graded supplementation of RSV (purity, 99%; 0, 0.5, 1.0, 2.5 and 5.0g/kg diet) to investigate the regulation of SIRT1 on lipid metabolism and antioxidant response (Supplementary Table 1). The juvenile fish with initial weight of 33.65±0.13g were randomly assigned to 15 tanks (800l) at a density of 30 individuals per tank, which were fed to apparent satiation twice daily (8:00 and 16:00) for 10weeks. At the end of the feeding experiment, ten fish were randomly selected from each tank, after 24h of starvation and anaesthetization with eugenol, to separate serum samples as described in Liu et al. (2021). Then, liver samples of four fish were separated for proximate composition and enzymatic activity analysis, and liver samples of the remaining six fish were isolated for gene expression and Western blot analysis.

### Triglyceride Content and Lipoprotein Content Analysis

The triglyceride (TG) content in liver and serum was analyzed spectrophotochemically at 546nm according to the colorimetric enzyme-linked TG detection method described by Schwartz and Wolins (2007). The content of serum cholesterol, low-density lipoprotein-cholesterol (LDL-C) and high-density lipoprotein-cholesterol (HDL-C) were measured with the commercial kits provided by Nanjing Jiancheng Bioengineering Institute (Nanjing, China).

### Hepatic Antioxidant Capacity Assay

The isolated liver samples of largemouth bass were homogenized in ice-cold phosphate buffer solution (1:10 dilution), and then centrifuged to separate the supernatant for the antioxidant capacity assay. The content of malondialdehyde (MDA) and hydrogen peroxide (H_2_O_2_), and activities of total antioxidative capacity (T-AOC), CAT and SOD were determined using commercial kits (Nanjing Jiancheng Bioengineering Institute, Nanjing, China). Hepatic soluble protein content was assayed following the description of Bradford (1976).

### Real-Time Quantitative PCR Analysis

The hepatic total RNA was extracted on ice with Trizol reagent (Takara, Japan) and then was reverse-transcribed into first-strand cDNA with the PrimeScript^™^ RT Regent kit (Takara). Real-Time Quantitative PCR (RT-qPCR) reactions were conducted in a BioRad CFX96 instrument using the SYBR Green Real-time PCR kit (Takara). The specific primes are listed in Table 1 and the procedure of PCR amplification was set as follows: 95°C for 2min, 40cycles of 95°C for 10s, 57°C for 10s, and 72°C for 20s, followed by a melting curve analysis. The gene β-actin was used as the house gene after its stability being verified, and relative expression of target genes involved in lipid metabolism and antioxidant response was calculated with the 2^-ΔΔCT^ method (Livak and Schmittgen, 2001).

### Western Blot Analysis

The isolated liver samples were homogenized in ice-cold RIPA buffer containing protease and phosphatase inhibitors (Beyotime Biotechnology, China) to extract total protein, which concentration was determined with the BCA Protein Assay Kit (Beyotime Biotechnology). The aliquots of protein samples (20μg) were separated by 10% SDS-PAGE gel and then transferred to activated polyvinylidene fluoride membranes (Merck Millipore, Germany). After being blocked with 5% skimmed milk in TBST buffer (Sangon Biotech, China), the membranes were incubated with the primary antibody overnight in a refrigerator at 4°C and then with secondary antibody for 2h. The immune complexes were visualized with a Beyo ECL Plus kit (Beyotime Biotechnology) and quantified using ImageJ software (Bethesda, United States). The specific primary antibodies are anti-FoxO1 (ET1608-25, HuaBio, China), anti-phospho-FoxO1 (#9461, Cell Signaling Technology, United States) anti-Akt1 (#4691, Cell Signaling Technology, United States), anti-phospho-Akt1 (#4060, Cell Signaling Technology, United States), anti-Sirt1 (sc-74,465, Santa Cruz Biotechnology), anti-Nrf2 (ab62352, Abcam, China) and anti-β-tubuliin (#2146, Cell Signaling Technology, United States) antibodies. The secondary antibodies are anti-mouse antibody (Solarbio, China) and anti-rabbit antibody (SongonBiotech, China).

### Statistical Analysis

All results were presented as mean ± standard error of the mean (SEM). All experimental data were statistically analyzed by one-way ANOVA in SPSS17.0 (IBM, America). Duncan’s multiple-range test was performed as multiple comparison test, and significance was considered as *p*<0.05.

## Results

### Cloning and Characterization of SIRT1

**Figure 1:**
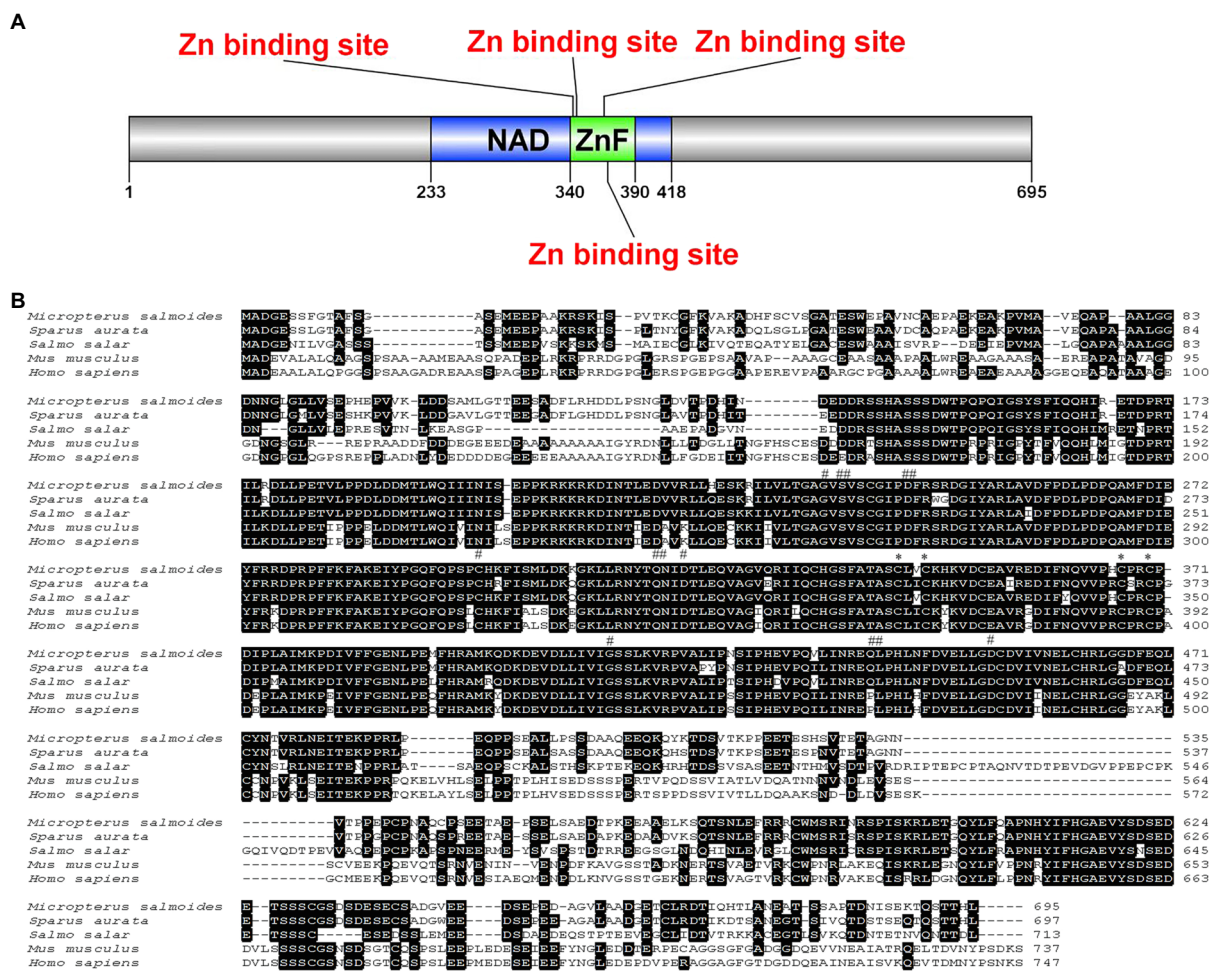
Schematic diagram prediction of largemouth bass SIRT1, including NAD^+^ binding domain, zinc ion binding module and unstructured region **(A)**. Multiple sequence alignment of SIRT1 with deduced amino acid sequences of other fish or mammal animals **(B)**, including *Sparus aurata* (AHX56273.1), *Salmo salar* (ENSSSAT00000005225.1), *Mus musculus* (NP_062786.1) and *Homo sapiens* (NP_036370.2). The predicted protein domain is indicated by horizontal line, and the substrate binding site, NAD^+^ binding site and Zn binding site are indicated by the “*”, “#” and “##” at the top of the sequence, respectively.

**Figure 2:**
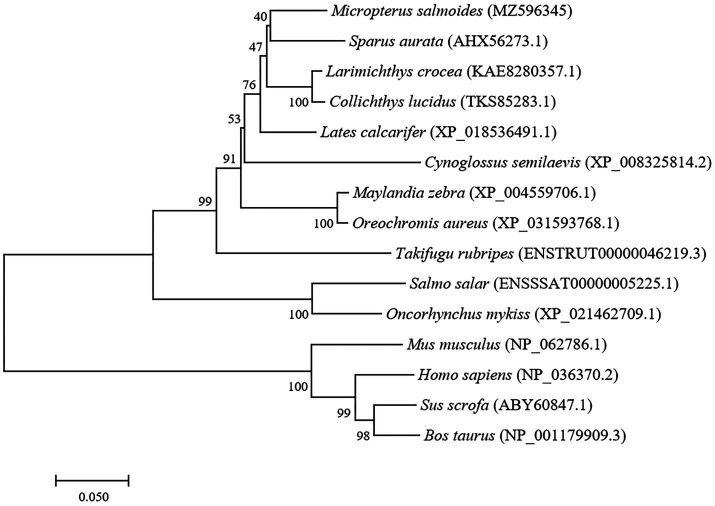
Phylogenetic tree of largemouth bass SIRT1 (GenBank No. MZ596345) with other vertebrate counterparts. The horizontal branch length is proportional amino acid substitution rate per site. The numbers represent the frequencies with which the tree topology presented here was replicated after 1,000 bootstrap iterations.

**Figure 3:**
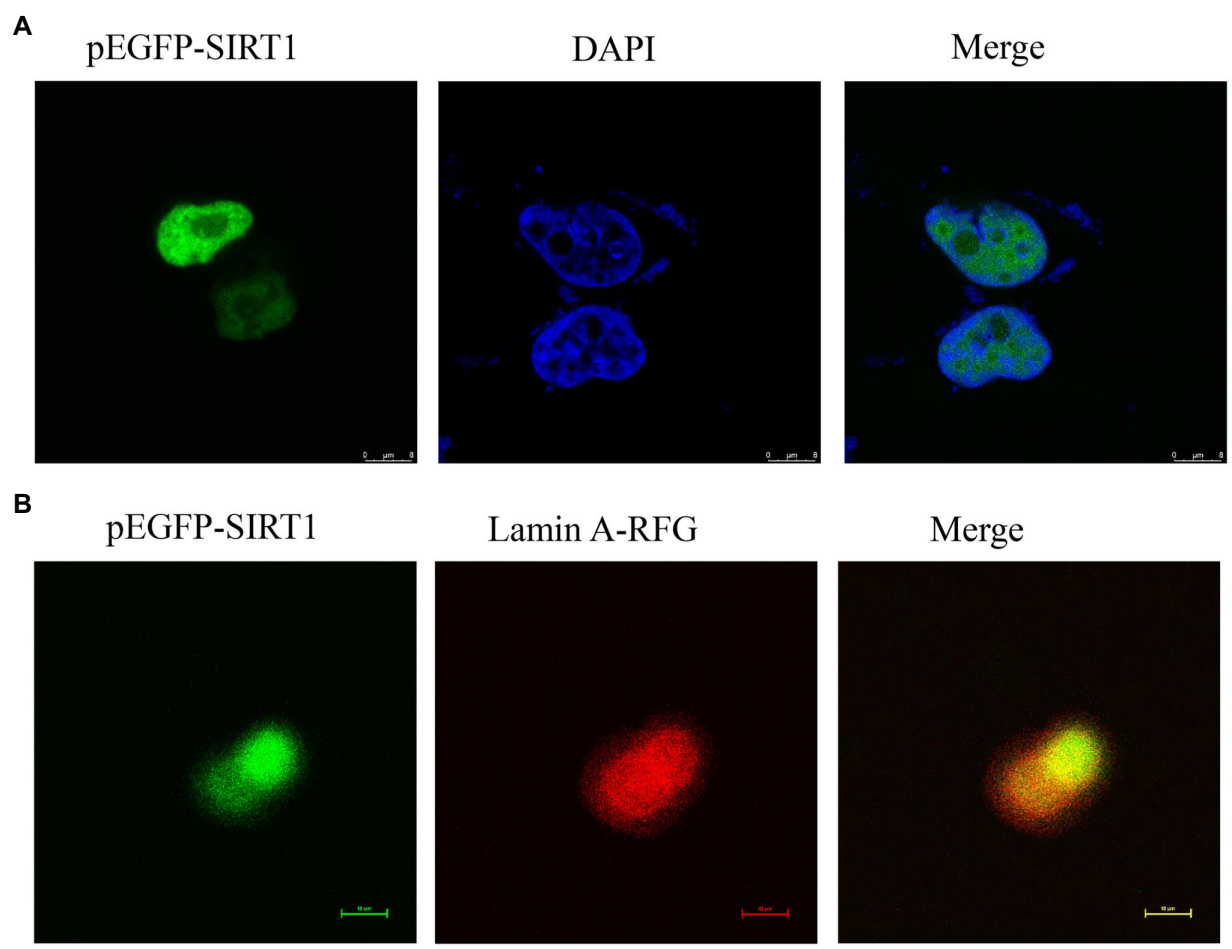
Localization of largemouth bass SIRT1 in HEK293t cells. **(A)** Localization of SIRT1-EGFP fusion protein, and the cells were fixed in 4% paraformaldehyde and nuclear labeled with DAPI were observed under a fluorescence microscope. **(B)** Localization of SIRT1-EGFP and Lamin A-RFP fusion protein.

The full-length cDNA of largemouth bass SIRT1 (GenBank No. MZ596345) was 3,395bp, which included a 46bp 5' untranslated region (UTR), a 1,261bp 3' UTR, and a 2088bp ORF encoding a polypeptide of 695 amino acids with a predicted molecular weight of 76.94 KDa, and a theoretical isoelectric point of 4.68. Blast analysis revealed that the deduced amino acids sequence of largemouth bass SIRT1 shared high identity with SIRT1 of other teleosts such as *Larimichthys crocea* (92.2%), *Sparus aurata* (90.7%) and *Lates calcarifer* (88.7%). The main functional domains of SIRT1 included the NAD^+^-binding domain and Zn-binding modules (Figure 1A). The above-conserved functional domains of SIRT1 in largemouth bass presented high homology with a variety of fish and humans (Figure 1B). Phylogenetic tree analysis revealed that largemouth bass SIRT1 clustered with SIRT1 of other teleosts, and more distantly with that of mammals (Figure 2). In addition, the subcellular localization analysis revealed that SIRT1-EGFP fusion protein in HEK 293t mainly targeted to the nucleus, labeled with DAPI or Lamin-RFP fusion protein (Figure 3).

### Tissue Expression of SIRT1

**Figure 4:**
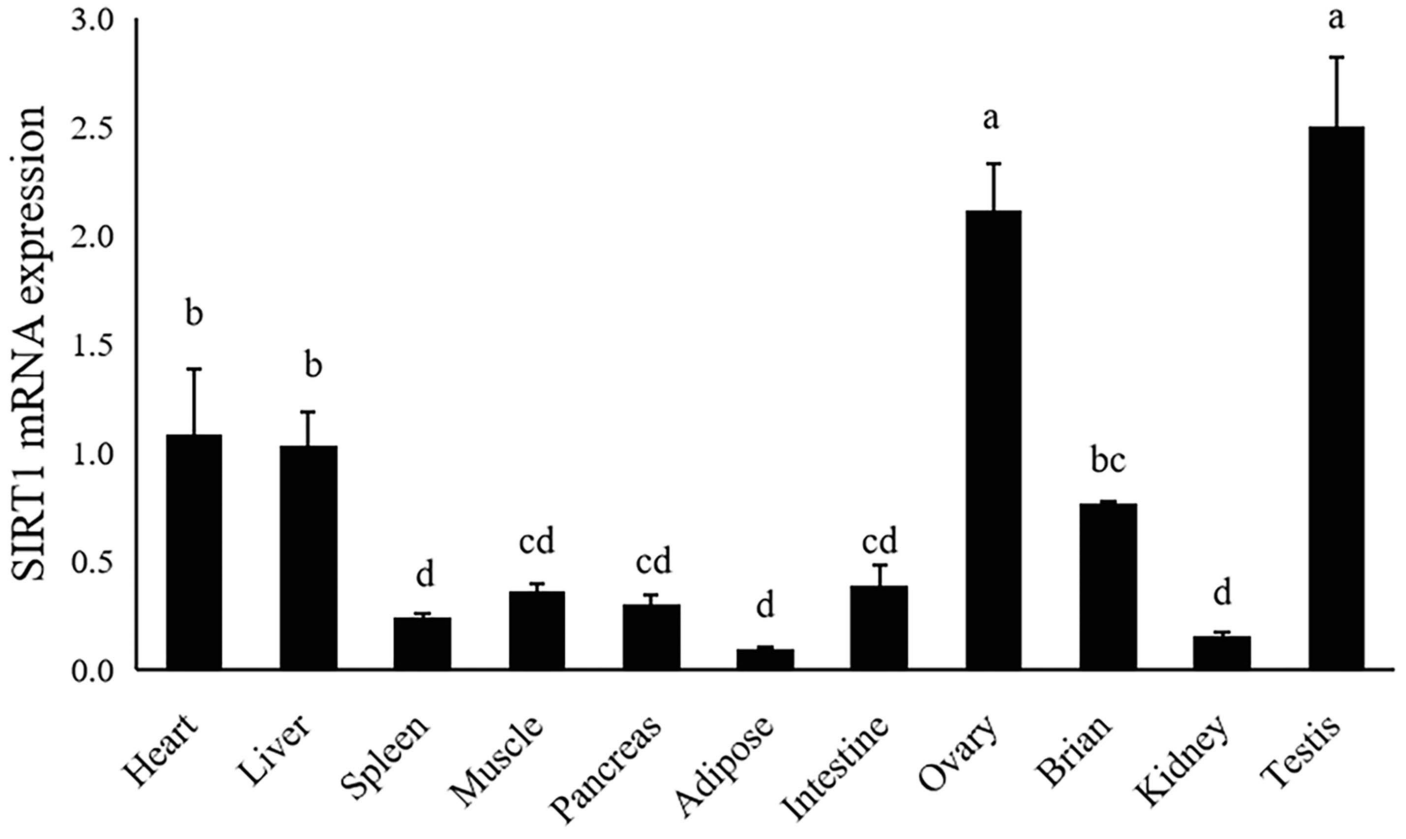
Tissue distribution of SIRT1 in largemouth bass. The same lower-case letters indicate no significant differences (*p*>0.05; Duncan’s test).

The gene expression of largemouth bass was ubiquitously expressed in all detected tissues including heart, liver, spleen, muscle, pancreas, adipose, intestine, ovary, brain, testis and kidney. The highest expression level of SIRT1 was observed in ovary and testis, moderate in liver and heart, and the lowest in spleen, kidney and adipose tissue (*p*<0.05; Figure 4).

### TG and Lipoprotein Content

Dietary supplementation of RSV significantly decreased the serum triglyceride (TG) content in a dose-dependent manner (*p*<0.05), and the lowest value was observed in the 5.0‰ (g/kg diet) RSV group (Table 2). The hepatic TG content followed the similar pattern with that of serum, and its content in fish fed diets with 2.5 and 5.0‰ RSV was significantly lower than that of 0.5‰ RSV group (*p*<0.05; Table 2). The inclusion of RSV did not produce any significant difference on the content of serum cholesterol (*p*>0.05; Table 2). Meanwhile, the content of serum LDL-C was significantly elevated with the supplementation of RSV (*p*<0.05), while the increased dose of RSV resulted in no statistical effect on its content (*p*>0.05; Table 2). Additionally, the fish fed the diet with 1.0‰ RSV obtained the highest HDL-C content, which was significantly higher than that without RSV inclusion (the control group; *p*>0.05; Table 2).

### Lipid Metabolism-Related Gene Expression

**Figure 5:**
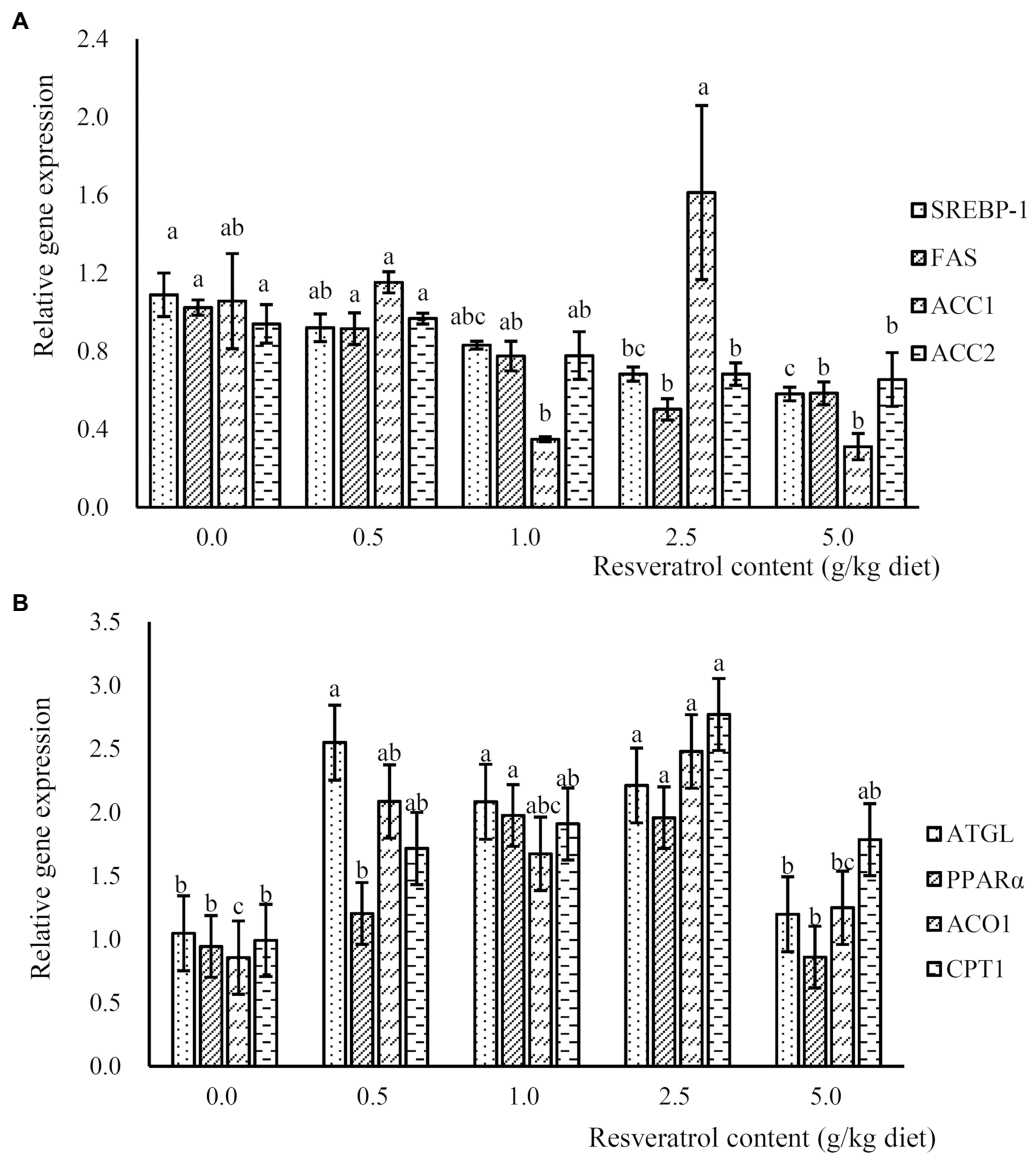
The expression of lipogenesis relates genes, SREBP-1, FAS, ACC1 and ACC2 **(A)**, and lipolysis related genes, ATGL, PPARα, ACO1 and CPT1 **(B)**, in response to graded levels of dietary RSV in largemouth bass for 10weeks. Values (means ± standard error of the mean, SEM) in bars that have the same letter are not significantly different (*p*>0.05; Duncan’s test) among treatments (*N*=3).

The expression of lipogenesis-related genes, such as sterol regulatory element-binding protein 1 (SREBP-1), fatty acid synthase (FAS) and acetyl CoA carboxylase-2 (ACC2), was significantly down-regulated in a dose-dependent manner with the increased RSV inclusion (*p*<0.05), and their expressions in fish fed the diet with 2.5 and 5.0‰ RSV were significantly lower than that without RSV inclusion (*p*<0.05; *p*<0.05; Figure 5A). However, no significant difference was observed in the expression of ACC1 among treatments (*p*>0.05; Figure 5A). The expression of adipose triglyceride lipase (ATGL) in fish fed diets with 0.5, 1.0 and 2.5‰ RSV was significantly higher than that of the control group (*p*<0.05; Figure 5B). Meanwhile, the inclusion of RSV elevated the expression of ACO1 (acyl-CoA oxidase), and its expression in fish fed diets with 0.5 and 2.5‰ RSV was significantly higher than the control (*p*<0.05; Figure 5B). The expression of carnitine palmitoyltransferase 1 (CPT1) followed a similar pattern with that of ACO1, and its expression in fish fed the diet with 2.5‰ RSV was significantly higher than the control group (*p*<0.05; Figure 5B).

### Antioxidant Capacity at Enzymatic and Transcriptional Level

The inclusion of RSV decreased the content of MDA, and its content in fish fed diets with 0.5, 1.0 and 2.5‰ RSV was significantly lower than the control group (*p*<0.05; Table 3). Meanwhile, the activity of SOD increased progressively with the supplementation of RSV, and its activity in fish fed diets with 0.5, 1.0 and 2.5‰ RSV was significantly higher than the control group (*p*<0.05; Table 3). The variation in T-AOC followed a similar pattern with that of SOD (Table 3). However, the activity of CAT decreased significantly as dietary RSV content increased, with the lowest value in the 2.5‰ RSV group (*p*<0.05; Table 3). Similarly, the lowest content of H_2_O_2_ was also observed in the 2.5‰ RSV group, which was significantly lower than that of 0.0, 0.5 and 1.0‰ RSV groups (*p*<0.05; Table 3).

**Figure 6:**
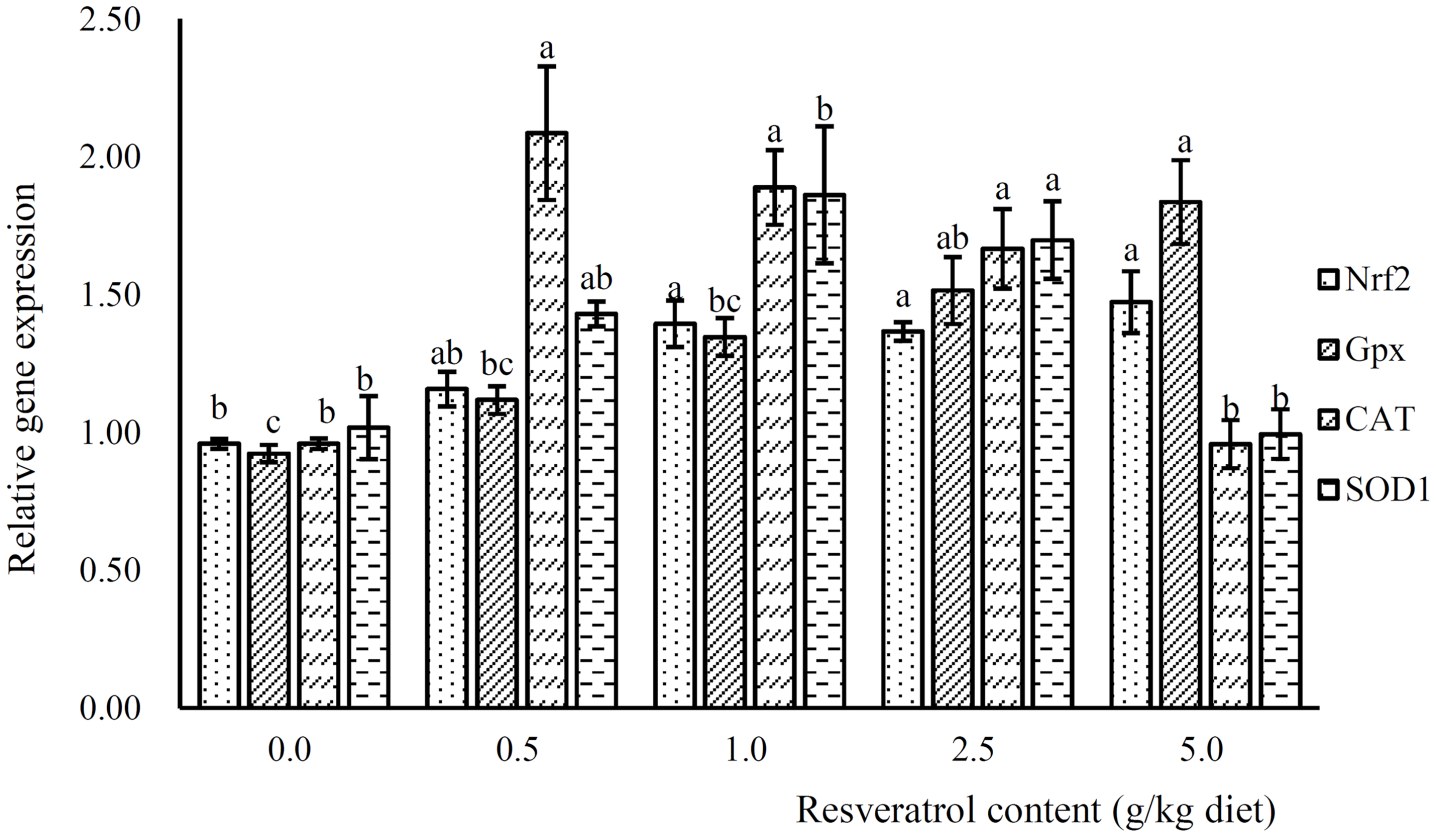
The expression of antioxidant related genes, Nrf2, Gpx, SOD1 and CAT, in response to graded levels of dietary RSV in largemouth bass for 10weeks. Values (means ± standard error of the mean, SEM) in bars that have the same letter are not significantly different (*p*>0.05; Duncan’s test) among treatments (*N*=3).

The expression of Nrf2 was significantly increased with the inclusion of RSV, and its expression was significantly higher in fish fed diets with 1.0, 2.5 and 5.0‰ RSV than the control group (*p*<0.05; Figure 6). Meanwhile, the inclusion of 0.5, 1.0 and 2.5‰ RSV significantly elevated the expression of CAT compared to the control group (*p*<0.05; Figure 6). The expression of SOD1 and SOD2 followed a similar pattern with that of CAT (Figure 6).

### Western Blot for SIRT1, Nrf2 and AKT1/FoxO1 Pathway

**Figure 7:**
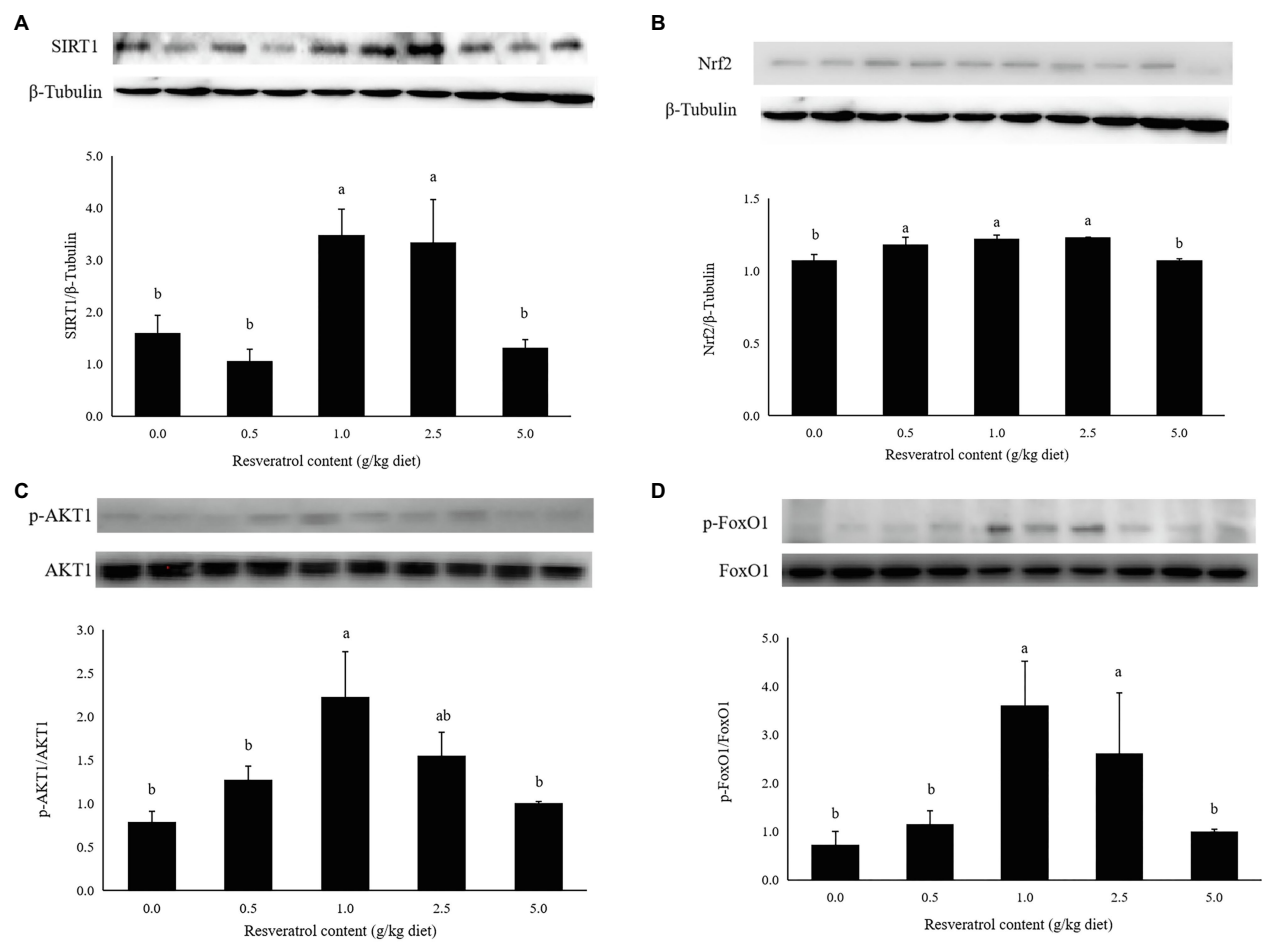
Effects of dietary RSV inclusion on SIRT1 (A), Nrf2 (B), p-FoxO1/FoxO1 (C), p-AKT1/AKT1 (D) protein expression in liver of largemouth bass. Values (means ± standard error of the mean, SEM) in bars that have the same letter are not significantly different (*p*>0.05; Duncan’s test) among treatments (*N*=3).

The protein expression of SIRT1 in fish fed diets with 1.0 and 2.5‰ RSV was significantly higher than the control group (*p*<0.05; Figure 7A). The inclusion of 0.5, 1.0 and 2.5‰ RSV significantly elevated the protein expression of Nrf2 compared to the control group (*p*<0.05; Figure 7B). The phosphorylation level of AKT1 was elevated with RSV inclusion, and the highest value was observed in fish fed the diet with 1.0‰ RSV, which was significantly higher than the control group (*p*<0.05; Figure 7C). The phosphorylation level of FoxO1 followed a similar variation trend with that of AKT1, and its phosphorylation level in fish fed diets with 1.0 and 2.5‰ diets was significantly higher than the control group (*p*<0.05; Figure 7D).

## Discussion

The abnormal lipid deposition and oxidative stress commonly impact the growth performance and health status of famed fish. In the present study, the full-length cDNA sequence of SIRT1 was cloned, which possessed the functions possibly related to the regulation of lipid metabolism and antioxidant capacity in mammals (Chong et al., 2012; Simmons et al., 2015). The functional domains analysis revealed that largemouth bass SIRT1 possessed the typical substrate-binding site and NAD^+^-binding domain, as in mammals (Davenport et al., 2014). In addition, the subcellular localization analysis revealed that largemouth bass SIRT1 was mainly in the nucleus, which was consistent with that in mammals (Houtkooper et al., 2012). The conserved characterization of SIRT1 between largemouth bass and mammals made it being a potential target for alleviating excessive lipid accumulation and oxidative stress. In mammals, SIRT1 can be activated by RSV, a natural polyphenol (Lagouge et al., 2006; Jiang et al., 2020). Previous studies have confirmed the role of RSV on SIRT1 activation in blunt snout bream (Zhang et al., 2018) and largemouth bass (Liu et al., 2021). In the present study, the inclusion of 1.0 and 2.5‰ RSV significantly elevated the protein expression of SIRT1, which was in accordance with its expression in the transcription level (Liu et al., 2021). Therefore, RSV was supplemented in the diets for largemouth bass to investigate the regulation of SIRT1 on lipid metabolism and antioxidant response in the present study. However, the inclusion of high dose RSV (5.0‰) produced no significant difference on the expression of SIRT1 protein compared to the control group, and the exact mechanism should be further explored.

The inclusion of RSV slightly reduced whole-body lipid levels of rainbow trout (Torno et al., 2019) and significantly alleviated the high-dietary carbohydrate-induced hepatic lipid accumulation of blunt snout bream (Shi et al., 2018). Consistently, the supplementation of RSV significantly decreased the triglyceride content in both liver and serum in the present study. In addition, the variation in content of serum cholesterol, LDL-C and HDL-C indicated that RSV inclusion significantly decreased the very low-density lipoprotein (VLDL), which content could be estimated by the subtraction of cholesterol content with the sum of LDL-C and HDL-C, further confirmed the alleviation role of RSV inclusion on lipid accumulation. SREBP-1 is known to play an important role in lipogenesis (Carobbio et al., 2013) and regulates genes involved in lipogenesis at the transcriptional level including FAS, which regulates the different steps of *de novo* fatty acid synthesis (Shao and Espenshade, 2012). In mammals, SIRT1 has been demonstrated to be involved in the regulation of hepatic lipogenesis through inhibiting the SREBP-1c activity (Ponugoti et al., 2010). In addition, RSV inclusion significantly down-regulated the expression of FAS in rat liver to prevent liver injury (Azirak et al., 2019), and the inhibitory effect of RSV on the expression of FAS was also observed in 3T3-L1 preadipocytes (Liang et al., 2013). In the present study, parallel with previous findings, the expression of SREBP-1 and lipogenesis-related genes, such as FAS and ACC2, was significantly down-regulated in fish fed the diet supplemented with RSV, and the inhibition of lipogenesis partly account for the decrease of TG content. ATGL plays an important role in the hydrolysis of triglycerides (Zimmermann et al., 2004). In the present study, the inclusion of 0.5, 1.0 and 2.5‰ RSV significantly increased the expression of ATGL, which was in accordance with the results in blunt snout bream (Zhang et al., 2018) and mammals (Lasa et al., 2012). Meanwhile, PPAR-α is a key mediator of lipid oxidation, which is involved in the regulation of fatty acid oxidation-related genes including ACO and CPT1 (Mandard et al., 2004). The positive correlation between the expression of PPARα and CPT1 has been observed in some fish species such as yellow catfish (Zheng et al., 2013), large yellow croaker (Ji et al., 2018) and nile tilapia (Zhang et al., 2019). It has been well demonstrated that SIRT1 can regulate lipid metabolism in mammals by positively regulating PPARα (Rodgers and Puigserver, 2007). In the present study, along with the promotion of SIRT1 protein expression induced by dietary RSV inclusion, the expression of CPT1 and ACO1 was remarkably increased. Consistently, the activation of PPARα in Nile tilapia significantly elevated the expression of CPT1 and ACO, and thus decreased the hepatic TG content (Ning et al., 2016). Therefore, the above finding may indicate that the activation of SIRT1 reduced hepatic lipid deposition through inhibiting lipogenesis and promoting lipolysis.

The regulation mechanism of SIRT1 on lipid metabolism has been well illustrated in mammals. SIRT1 has a negative regulatory effect on lipid accumulation through the Akt/FoxO1 pathway (Tobita et al., 2016), and the transcriptional factor, FoxO1, is a downstream factor of SIRT1 and plays an important role in the regulation of lipid metabolism (Gross et al., 2008; Sparks and Dong, 2009). It has been well demonstrated that the activation of FoxO1 inhibits the expression of lipogenic genes including SREBP-1c (Zhang et al., 2006), and reduced fatty acid oxidation in mammals (Matsumoto et al., 2006). In the present study, the activation of SIRT1 induced by dietary RSV inclusion led to the phosphorylated activation of AKT1 and phosphorylated inhabitation of FoxO1 in the 1.0‰ RSV and 2.5‰ RSV groups, and this indicated that the AKT1/FoxO1 pathway was involved in the alleviation of hepatic lipid deposition thorough the regulation of lipogenesis and lipolysis as in mammals.

The stress response, oftentimes induced by environmental factors, artificial feeds and some other human factors, commonly resulted in contaminant-stimulated reactive oxygen species which mainly leads to oxidative damage such as lipid peroxidation and DNA damage (Chowdhury and Saikia, 2020). In addition, oxidative stress plays a central role in the pathogenesis of nonalcoholic fatty liver disease in mammals (Chen et al., 2020). Therefore, the elevation of antioxidant capacity may partly benefit for the health maintenance and lipid deposition alleviation. In the present study, the content of MDA, a product of lipid peroxidation, was significantly decreased, while the activity of antioxidant enzymes, such as SOD and T-AOC was elevated significantly. This indicated that the inclusion of RSV significantly elevated antioxidant capacity of largemouth bass, and similar results have been observed in turbot (Tan et al., 2019) and blunt snout bream (Jia et al., 2019). However, the activity of CAT was significantly depressed with the inclusion of RSV, which converts H_2_O_2_ to oxygen and water. Meanwhile, the content of H_2_O_2_ decreased significantly with the RSV inclusion, and this may indicate that CAT is a substrate-dependent enzyme. The variation in enzymatic activity of antioxidant enzyme commonly consistent with that in transcriptional level (Zheng et al., 2016; Li et al., 2020). Consistently, the relative expression of SOD1, CAT and Gpx was significantly elevated with the inclusion of 0.5, 1.0 and 2.5‰ RSV, which followed a similar pattern with the expression of SIRT1. Nuclear factor E2-related factor 2 (Nrf2) is a key nuclear transcription factor, which can regulate genes containing ARE, and plays an important role in the transcription of antioxidant genes in mammals (Alam et al., 1999; Kensler et al., 2007). In previous study in mammals, it has been manifested that SIRT1 plays an antioxidant role by activating Nrf2/antioxidant response element (ARE) pathway (Ding et al., 2016). In this study, the protein expression of NRF2 and SIRT1 is positively correlated, and the mRNA expression of Nrf2 is directly proportional to antioxidant-related genes SOD1 and Gpx, which was similar to the results obtained in zebrafish (Timme-Laragy et al., 2009), Jian carp (Wu et al., 2014) and blunt snout bream (Yu et al., 2019). Therefore, the potential regulation of SIRT1 on antioxidant genes was mediated by Nrf2.

## Conclusion

The characterization of largemouth bass SIRT1 is relatively conserved with mammals. The activation of SIRT1 induced by dietary RSV supplementation significantly alleviated hepatic lipid accumulation partly mediated by the AKT1/FoxO1 pathway, and elevated the antioxidant capacity at both transcriptional and enzymatic level with the regulation of Nrf2. The above results confirmed the positive role of SIRT1 in alleviating hepatic lipid deposition and oxidative stress in largemouth bass. Therefore, in practice, the advantage of SIRT1 activation, which can be achieved by nutritional or genetic methods, should be used to improve the health status of largemouth bass, and this can further advance the culture of largemouth bass and other aquaculture species.

## Data Availability Statement

The datasets presented in this study can be found in online repositories. The names of the repository/repositories and accession number(s) can be found in the article/Supplementary Material.

## Ethics Statement

The animal study was reviewed and approved by the Animal Care and Use Committee of the Shanghai Ocean University.

## Author Contributions

YH: investigation, formal analysis, and writing - original draft. SW: investigation methodology and data curation. XM: investigation methodology. NC: conceptualization, project administration, and funding acquisition. SL: conceptualization, supervision, writing – review and editing, and funding acquisition. All authors contributed to the article and approved the submitted version.

## Funding

All sources of funding received for the research has been submitted. Briefly, this work was financially supported by “Chenguang program” supported by Shanghai Education Development Foundation and Shanghai Municipal Education Commission (19CG56), China Agriculture Research System of MOF and MARA (CARS-46), National Natural Science Foundation of China (31802308), and Shanghai Talent Development Fund (2019097).

## Conflict of Interest

The authors declare that the research was conducted in the absence of any commercial or financial relationships that could be construed as a potential conflict of interest.

## Publisher’s Note

All claims expressed in this article are solely those of the authors and do not necessarily represent those of their affiliated organizations, or those of the publisher, the editors and the reviewers. Any product that may be evaluated in this article, or claim that may be made by its manufacturer, is not guaranteed or endorsed by the publisher.

## Footnotes

^1^ http://www.ncbi.nlm.nih.gov/blast

^2^ https://www.ncbi.nlm.nih.gov/Structure
