# Supplementary material for: Molecular Cloning and Characterization of Sirtuin 1 and Its Potential Regulation of Lipid Metabolism and Antioxidant Response in Largemouth Bass (Micropterus salmoides)
Source: Front Physiol. 2021 Sep 7;12:726877. doi: 10.3389/fphys.2021.726877 (PMC8504536; doi:10.3389/fphys.2021.726877)
Supplement: Supplementary file 1 [file Data_Sheet_1.docx]

**Supplemental Table 1** Formulation and chemical composition of experimental diets (g/kg diet).

| Ingredients | Diets | | | | | |
| --- | --- | --- | --- | --- | --- | --- |
|  |  | NC | RSV (0.5) | RSV (1.0) | RSV (2.5) | RSV (5.0) |
| Resveratrol^*^ |  | 0.0 | 0.5 | 1.0 | 2.5 | 5.0 |
| Microcrystalline cellulose |  | 10.0 | 9.5 | 9.0 | 7.5 | 5.0 |
| White fish meal^†^ |  | 420.0 | 420.0 | 420.0 | 420.0 | 420.0 |
| Shrimp meal^†^ |  | 60.0 | 60.0 | 60.0 | 60.0 | 60.0 |
| Blood meal^†^ |  | 30.0 | 30.0 | 30.0 | 30.0 | 30.0 |
| Wheat gluten meal^†^ |  | 30.0 | 30.0 | 30.0 | 30.0 | 30.0 |
| Fermented soybean meal^†^ |  | 90.0 | 90.0 | 90.0 | 90.0 | 90.0 |
| Corn gluten meal^†^ |  | 110.0 | 110.0 | 110.0 | 110.0 | 110.0 |
| Squid viscera meal^†^ |  | 20.0 | 20.0 | 20.0 | 20.0 | 20.0 |
| Brewer’s yeast meal^†^ |  | 20.0 | 20.0 | 20.0 | 20.0 | 20.0 |
| Soybean oil |  | 50.0 | 50.0 | 50.0 | 50.0 | 50.0 |
| Soybean Lecithin |  | 25.0 | 25.0 | 25.0 | 25.0 | 25.0 |
| Vitamin mixture^‡^ |  | 10.0 | 10.0 | 10.0 | 10.0 | 10.0 |
| Mineral mixture ^§^ |  | 10.0 | 10.0 | 10.0 | 10.0 | 10.0 |
| Ca(H_2_PO_3_)_2_ |  | 10.0 | 10.0 | 10.0 | 10.0 | 10.0 |
| α-Starch |  | 100.0 | 100.0 | 100.0 | 100.0 | 100.0 |
| Zeolite powder |  | 0.0 | 0.0 | 0.0 | 0.0 | 0.0 |
| Cr_2_O_3_ |  | 5.0 | 5.0 | 5.0 | 5.0 | 5.0 |
| Proximate analysis (%, on a dry weight basis) | | | | | | |
| Crude protein |  | 534.0 | 533.5 | 532.8 | 532.3 | 531.9 |
| Crude lipid |  | 86.6 | 89.6 | 89.3 | 93.8 | 89.4 |
| Crude fiber |  | 17.7 | 16.7 | 16.4 | 15.8 | 14.0 |
| Digestible starch |  | 104.5 | 109.1 | 105.1 | 107.1 | 110.0 |
| Ash |  | 122.2 | 119.5 | 123.1 | 119.6 | 120.0 |
| Gross energy (MJ/kg) |  | 191.4 | 192.6 | 191.9 | 193.7 | 192.9 |

^*^ Acquired from Xi’an Quan’ao Biotech Co., Ltd (Shanxi, China), purity, 99%.

^†^ Supplied by Xinxin Tian’en Feed Corporation (Zhejiang, China): white fish meal, crude protein, 72.01%, crude lipid, 7.46%; shrimp meal, crude protein, 61.72%, crude lipid, 3.62%; wheat gluten meal, crude protein, 80.05%; fermented soybean meal, crude protein, 51.67%, crude lipid, 2.62%; corn gluten meal, crude protein, 60.41%, crude lipid, 5.12%; squid viscera meal, crude protein, 49.27%, crude lipid,19.07%; brewer’s yeast meal, crude protein, 37.05%, crude lipid, 0.52%;

^‡^ Vitamin Premix (mg/kg diet): vitamin A, 16000 IU; vitamin D_3_, 8000 IU; vitamin K_3_, 14.72; vitamin B_1_, 17.80; vitamin B_2_, 48; vitamin B_6_, 29.52; vitamin B_12_, 0.24; vitamin E, 160; vitamin C, 800; niacinamide, 79.20; calcium-pantothenate, 73.60; folic acid, 6.40; biotin, 0.64; inositol, 320; choline chloride, 1500; L-carnitine, 100.

^§^ Mineral Premix (mg/kg diet): Mineral Premix (mg kg^−1^ diet): Cu (CuSO_4_), 2.00; Zn (ZnSO_4_), 34.4; Mn (MnSO_4_), 6.20; Fe (FeSO_4_), 21.10; I (Ca(IO_3_)_2_), 1.63; Se (Na_2_SeO_3_), 0.18; Co (CoCl_2_), 0.24; Mg (MgSO_4_·H_2_O), 52.70.

**Supplemental Fig. 1** Nucleotide and deduced amino acids sequences of largemouth bass SIRT1.

1 TGCAAATGAACCAGCTGTCCGAGAGGAAGGACCTGCTGGGGCGAAGATGGCGGACGGAGA

1 M A D G E

61 GAGCAGTTTCGGAACGGCTTTTTCAGGCGCATCCGAAATGGAAGAACCTGCCGCGAAAAG

21 S S F G T A F S G A S E M E E P A A K R

121 GTCGAAAATCAGTCCGGTGACTAAATGCGGATTCAAAGTCGCCAAAGCAGATCATTTCTC

41 S K I S P V T K C G F K V A K A D H F S

181 ATGCGTCTCCGGGGCTACGGAGAGCTGGGAGCCGGCGGTGAATTGTGCGGAGCCAGCGGA

61 C V S G A T E S W E P A V N C A E P A E

241 GAAGGAAGCAAAGCCGGTGATGGCGGTAGAGCAGGCCCCAGCGGCGCTAGGCGGAGACAA

81 K E A K P V M A V E Q A P A A L G G D N

301 CAATGGACTGGGACTGCTGGTCTCCGAGCCGCACGAACCAGTTGTGAAACTAGACGACAG

101 N G L G L L V S E P H E P V V K L D D S

361 CGCTATGCTTGGAACAACTGAGGAGAGTGCTGATTTTCTTAGACATGACGACCTCCCCTC

121 A M L G T T E E S A D F L R H D D L P S

421 AAACGGGCTGGATGTAACACCGGACCACATCAATGACGAAGATGACAGATCCTCACATGC

141 N G L D V T P D H I N D E D D R S S H A

481 AAGCTCCAGCGACTGGACTCCTCAACCGCAAATAGGGTCCTACAGTTTCATCCAGCAACA

161 S S S D W T P Q P Q I G S Y S F I Q Q H

541 CATCAGAGAGACCGATCCAAGGACCATTCTGAGGGACTTGCTGCCCGAGACTGTACTCCC

181 I R E T D P R T I L R D L L P E T V L P

601 ACCAGATTTAGACGACATGACATTGTGGCAGATCATCATTAACATCTCAGAACCTCCGAA

201 P D L D D M T L W Q I I I N I S E P P K

661 AAGAAAGAAGCGGAAGGATATCAACACCTTAGAAGATGTGGTCAGACTACTCCATGAAAG

221 R K K R K D I N T L E D V V R L L H E S

721 TAAAAGGATCCTTGTGCTGACTGGTGCTGGGGTGTCAGTTTCATGTGGAATACCAGACTT

241 K R I L V L T G A G V S V S C G I P D F

781 TCGCTCCAGAGATGGGATTTATGCACGGCTTGCTGTAGATTTTCCTGATCTTCCAGACCC

261 R S R D G I Y A R L A V D F P D L P D P

841 TCAAGCTATGTTTGACATTGAATACTTCAGACGAGACCCAAGACCCTTTTTCAAGTTTGC

281 Q A M F D I E Y F R R D P R P F F K F A

901 TAAGGAGATCTACCCTGGTCAGTTTCAGCCTTCACCATGTCACAAATTCATATCTATGCT

301 K E I Y P G Q F Q P S P C H K F I S M L

961 GGATAAGAAGGGGAAGCTGCTGCGCAATTATACACAAAACATTGATACATTAGAACAAGT

321 D K K G K L L R N Y T Q N I D T L E Q V

1021 GGCTGGAGTCCAGCGGATTATTCAGTGCCATGGGTCGTTTGCAACTGCATCCTGTCTGGT

341 A G V Q R I I Q C H G S F A T A S C L V

1081 CTGTAAACACAAAGTGGATTGTGAGGCTGTAAGGGAAGACATCTTTAACCAGGTTGTCCC

361 C K H K V D C E A V R E D I F N Q V V P

1141 TCATTGTCCACGGTGTCCAGATATTCCCCTGGCAATCATGAAACCTGACATCGTCTTCTT

381 H C P R C P D I P L A I M K P D I V F F

1201 TGGCGAGAACCTTCCAGAAATGTTCCACAGAGCCATGAAGCAGGATAAAGATGAGGTGGA

401 G E N L P E M F H R A M K Q D K D E V D

1261 CCTCTTGATTGTCATTGGTTCTTCACTTAAAGTCCGACCAGTTGCCCTCATCCCAAACTC

421 L L I V I G S S L K V R P V A L I P N S

1321 CATTCCTCATGAAGTGCCTCAGGTCCTGATCAATAGAGAGCAGCTGCCACACCTAAACTT

441 I P H E V P Q V L I N R E Q L P H L N F

1381 TGATGTGGAGTTGCTTGGGGACTGTGATGTCATTGTCAACGAGCTCTGTCATCGTTTGGG

461 D V E L L G D C D V I V N E L C H R L G

1441 TGGAGACTTTGAGCAGCTCTGCTACAACACTGTAAGACTCAATGAGATCACAGAGAAGCC

481 G D F E Q L C Y N T V R L N E I T E K P

1501 CCCTCGGTTACCAGAACAGCCACCAAGTGAGGCCTTGCTTCCTTCTAGCGATGCAGCTCA

501 P R L P E Q P P S E A L L P S S D A A Q

1561 GGAGGAGCAGAAGCAGTACAAAACAGACTCGGTAACGAAGCCTCCAGAGGAGACAGAAAG

521 E E Q K Q Y K T D S V T K P P E E T E S

1621 TCACAGTGTCACGGAGACTGCTGGTAATAATGTTACACCTCCAGAGCCTTGTCCAAATGC

541 H S V T E T A G N N V T P P E P C P N A

1681 TCAGTGTCCCAGTGAAGAGACGGCTGAGCCTTCAGAGTTATCAGCAGAAGACACACCAAA

561 Q C P S E E T A E P S E L S A E D T P K

1741 GGAAGAAGCCGCCGAATTAAAGAGCCAAACCTCCAACCTTGAATTCCGTAGACGATGCTG

581 E E A A E L K S Q T S N L E F R R R C W

1801 GATGAGTCGAATCAACAGAAGTCCAATCAGCAAACGCCTTGAGACAGGCCAGTACCTGTT

601 M S R I N R S P I S K R L E T G Q Y L F

1861 TCAAGCACCAAATCACTATATCTTCCATGGGGCGGAGGTTTACTCTGACTCTGAAGATGA

621 Q A P N H Y I F H G A E V Y S D S E D E

1921 GACGTCGAGCTCCTGTGGGAGTGACAGTGACGAGTCTGAATGCAGTGCAGATGGGGTGGA

641 T S S S C G S D S D E S E C S A D G V E

1981 AGAAGACAGCGAGCCGGAGGACGCCGGCGTACTAGCAGCGGATGGAGAAACATGCCTCAG

661 E D S E P E D A G V L A A D G E T C L R

2041 AGACACAATACAACACACTTTAGCCAATGAGGCCACATCAAGTGCGCCGACAGACAATAT

681 D T I Q H T L A N E A T S S A P T D N I

2101 TTCTGAAAAAACTCAGAGCACCACACACCTTTAAATGTAAAGTTGACAAAGCACTAATTG

701 S E K T Q S T T H L *

2161 TCTTTTTTTTTTATATATATATATATATATATATATATAATAATGTGTTTCATATATTTT

2221 TGACTCATCAACTTTACTATTATTTTTCATAAAGCTTAAATTGCGTGACAACTTGTGTGT

2281 GAATATGCAGGAAGTTATTTTTTTCCTCTTTTTTTGTTTTTCTTTTTGTGACCAGTTTGG

2341 TAACACACATTGTGTCTGAAGCCTCATACATGTATACTACTTACTAAATAGAATGCAGTT

2401 TTTCTTTTTTTTTTAGTATTTCTTTCCATATGCTCACTTCATTTTGCATTCACATTTTCA

2461 GAAATGTAGCTGATGCTCTCATCTACTACGGCATACCATGAATTCAGCACTAGATTGAGC

2521 TTTAATTCCTTAATTGCAGTCATTACAAGCAAAGACATCGGATGTCTTAGTCCATAGTGT

2581 GATGTCAGCTGTGGTCTAATGGTGTGTTTTAGAGTTGGTCCATGTTAACATTGGGAAATG

2641 TGTAAATATGTATGCCTACTGCTTGCTAAATAGTGGGTACTATGAGTTTCAGACACATAC

2701 ATAAATCTTAAAATGGTTTACTTGAACGCATTATCTAACTTGCAATATAAAAAGCTCTGC

2761 TGCCTTTTCATGTGGGCAAATGGTGGAAGAACAGTACTCTTTAAGTTTAGACCAAAGAAC

2821 ACTTTATCAACCCGCCCTAAGTTGTCATAAGCTATGTATGTTTAAACTGTCATTTAAGTC

2881 TTGGAGAAAAATACAACTATTGACAAGGTGTTCCTTCTGACATGTGGCAGCTACTTTTTA

2941 TGGTCAAGCCTTTTTATTTTTATCCACCGATGTGAAACGTACTCATGAAAGAGTAGTAGT

3001 TTGTATCTGTACAAAGTTAAACTAAGTTGTAATATTAACCCTTAGGAATATATTCAGGGA

3061 CTTCGTATTGGAAAATGGCCCTTTTCCCCCTTCATATAAAACCAGTTTTGTTGCCCATGT

3121 CATTTTTCATGGAATATAAAGTTAAACCAGTTTATTTTAGAGGAGTCGGCTGCCTGTAAA

3181 GACGTGTTGGTAGATTATTTGAGCCAATGTGACAGATAAATGTCCCCCCCAACATAATTC

3241 ACATACAAGGTAATGTTTTGTTTCCGGTTTAATGAAATATGATTTTTTTTTTTTTTACCC

3301 CATATTAGTTGTTGTCTTTTCTGGGGTTCTCAGTAAAACAAGAATGAACAAAAAAAAAAA

3361 AAAAAAAAAAAAAAAAAAAAAAAAAAAAAAAAAAA
